# Diphtheria

**Published:** 1884-01

**Authors:** 


### DIPHTHERIA.

From a lecture by Dr. Morrell Mackenzie, published in *Med. Record*, we extract the following:

I think that, at the beginning, diphtheria is a local disease. I believe that the effect of the poison may sometimes be so great that the disease appears to be constitutional from the commencement. I believe that such cases are analogous to those of scarlet fever or small-pox, where the patient is struck down at the very moment of the invasion of the disease. The poison must enter through some part of the system, and I believe that it is local at the beginning. These points bear upon prognosis, and are of great importance. From prognosis we will now pass to

### TREATMENT.

Here, again, remedies of the most varied character possible have been recommended. I recollect reading a paper written by a French physician, in which he said he bled every patient, and that he treated fifty or sixty, and every one recovered. All I

can say is that if we should treat diphtheria in London in this way, I think we would almost be prosecuted. It is exceedingly bad treatment. It only shows that it is possible to make a bad diagnosis, or else it is possible for some people to stand depletion in a most extraordinary manner.

The first great point in the treatment of this disease is to attend to constitutional measures and then to local treatment. The constitutional treatment is of no less importance than the local. It is necessary to support the patient from the beginning, and stimulants are of the utmost importance. Do not wait until the patient becomes depressed, but give stimulants from the very commencement. This is an exception to all diseases, and you must begin with stimulants at the commencement, and give them in the more solid form, such as brandy diluted with water, or port wine; such as furnish nutriment as well as alcohol. When the patient is beginning to recover, the light wines, especially champagne, are useful; but, in the early stages, port wine, with water, is one of the most useful you can give.

Stimulants must be given during the night as well as during the day in a very large number of cases. I have seen many cases where patients have died through want of having stimulants administered during the night. In young children it is very frequently necessary to awaken the patient and give stimulants. As a general rule, it is bad to wake a patient out of a refreshing sleep to give medicines; but here is an exception, and I would say that if the child sleeps more than four hours, it must be awakened and stimulants and nourishment administered.

We now pass on from the use of stimulants to the use of medicines. Here, again, we meet with a very great variety, but the most useful, perhaps, of all, is the perchloride of iron. In this matter I am entirely in accord with Professor Jacobi, who has found the remedy more useful than any other. Professor Jacobi has laid it down that this medicine should be given in full doses. It is also important to give a per salt of iron, which can be assimilated with comparative ease, and probably the perchloride is the best you can use, and of it at least a drachm a day, diluted with water, should be administered; fifteen drops, well diluted with water, four times a day. The only time when I have not given the perchloride of iron has been when I have been trying the local effects of some agent that has been employed. Quinine is a very useful medicine. When the temperature is high it has a very great effect in bringing it down nearly or quite to the normal. These are perhaps the most important of the constitutional remedies.

All sorts of specifics have been recommended, but I have not had much success with them. Chlorate of potash has been very much praised, both as a constitutional and a local medicine. You may give it, because it cannot, in proper doses, do much harm, and it may do some good. There is one remedy which has been recommended by a gentleman whom I see before me, Dr. Beverley Robinson, and that is copaiba, which has an important effect upon mucous membranes, as possibly some of you may have had occasion to observe. But its effects are not confined to the mucous membrane of the urethra. It also produces a marked effect upon the mucous membrane of the pharynx and larynx, and that of the whole bronchial tract. I have tried Dr. Robinson's recommendation, giving the medicine in the form of pearls, which the French make, and which children take very easily, and I have administered them with great success. But I must mention that I have used it in the catarrhal form of diphtheria—the milder cases where the exudation is not very adhesive. When the more serious cases of diphtheria are about, you get a large number of cases of catarrhal diphtheria, and in those you will find great benefit following the administration of copaiba.

We will next pass to local remedies, and here again we have a very wide field. A great many doctors may go through a lifetime and see only a few cases of diphtheria. Some meet with severe epidemics, and others with epidemics mild in character. The consequence is that an immense number of remedies are not only recommended, but the doctors say that they have not lost a case since they began to use such and such remedies. You must look upon such statements with great suspicion, and it is safe to consider that the doctors who have treated so large a number of cases with such uniform success have, at least, treated a mild type of diphtheria.

The local remedies in most common vogue are lime-water and lactic acid. Both of these remedies have one great advantage; they do not do any harm, and here I may say, gentlemen, that it is a great thing, when you are trying a remedy, to use one that does no harm. In earlier days severe caustics were used, such as hydrochloric acid, nitrate of silver, and, if the patient recovered, it was always thought that event was due to the acid or the silver. But all that has been changed. We now know that if strong caustics are used the effect is almost always to cause extension of the disease. The remedy inflames and irritates, and a false membrane is formed in close contiguity to that which previously existed. When we were suddenly told by German physicians that lactic acid was used with great benefit, and also lime-water, the news was so gratifying that we all used these remedies, which were not injurious or painful to the patient. Both have been found useful.

I ought to say here that certain solutions have been said to be useful because of the effect they produce upon the false membrane, causing it to gradually dissolve and disappear in a short time. But, unfortunately, when we have to deal with the living subject we have a totally different condition of things from that which is present in making experiments, and I have found that when using substances locally sufficient to have any effect upon the false membrane, they had an irritating effect on the mucous membrane which I was treating. Hence I returned to the use of such remedies as do not irritate, and have given up those which had a reputation for dissolving false membrane. With regard to lactic acid and lime-water, they do not have much effect upon the false membrane in the test-tube, but they certainly do seem to have considerable effect when applied to false membrane growing upon mucous membrane. It is very difficult to make accurate observations with regard to the progress of the disease from hour to hour in children; but I have had opportunity to try both remedies upon false membrane inside of the lip and upon the tongue, where I could watch the effect. I recollect three cases in which I tried the experiment with lime-water where false membrane was growing upon the inside of the lip. I treated one side with lime-water and left the other to nature, and the side treated rapidly improved, while the other remained stationary. So I believe that lime-water is useful as a local application, and in this respect I differ with my friend Dr. Jacobi, who believes that both lactic acid and lime-water have been over-estimated. I strongly recommend that you should use them in every case.

We now pass on to another class of remedies, which I wish to bring to your notice, namely, those which shut out the air. This class of remedies I have introduced, and they have been employed in England to some extent. I refer to what may be called varnishing the mucous membrane with benzoin, or tolu dissolved in ether or chloroform or alcohol, and also used in various mixtures. I found as the result of considerable experiment that tolu dissolved in ether in the proportion of 1 to 5, made an excellent varnish, and that when applied to the mucous membrane it did not cause pain or inconvenience, was sufficiently strong to hold, and did not require to be repeated. Many of these local remedies have been recommended on the ground that they destroy germs. Just here it occurs to me that I have omitted to speak of carbolic acid and salicylic acid, etc. Carbolic acid is an excellent remedy, and it has the effect, as has been demonstrated, of destroying germs, and if used sufficiently diluted it will do no harm.

All this class of remedies have been recommended upon the scientific ground that they destroy germs.

The principle upon which I have introduced the remedies which varnish the mucous membrane is, that whatever the poisonous element may be, whether a vegetable growth or some other germ, or something else, this living matter that causes false membrane to be formed, requires the presence of air. Directly you exclude the air you prevent the growth of germs which require air for their existence. As soon as possible, therefore, I apply this varnish over the false membrane; not only over the false membrane, but all around it. It is of itself to a certain extent a germ destroyer, but everything depends upon the coating of varnish being air-tight. Some of my friends, at first, found considerable difficulty in applying it, and I also had the same experience. At first I wiped the surface, to which it was to be applied, with blotting-paper. I carefully applied this absorbing material to different parts of the throat, and then immediately afterwards applied this varnish. This plan answers perfectly well when you can do it; but every now and then you will find a patient who will retch a little just after the blotting-paper has touched the surface, and the mucous membrane becomes wet before you can apply the varnish. I then adopted the plan of putting a piece of lint around my finger and drying the throat with this, and then quickly applying the varnish with a brush. This does not hurt the child, and I speak of children because nine-tenths of our cases occur among children, and it answers perfectly well; but if you should have difficulty with this, I should advise you to apply the varnish all the same. I have had several patients treated entirely by the use of varnish, without constitutional remedies, and with good results.

I shall feel exceedingly proud if, as the result of this lecture, gentlemen shall try the effect of this varnish.

I will now say a few words with reference to the use of steam and the use of ice. Both these remedies are useful, but they should be applied in different classes of cases. In the early stages it is very useful to employ ice. It affords the greatest comfort to the patient. Let them have ice, and take as much as possible. Many young children are pleased to have pieces of ice put into their mouths. There is no doubt that it restricts the associated inflammation so often present. In the early stages it is most desirable to use ice, and you can use any amount of it without doing harm. It is only in exceptional cases, where the patient is very much depressed, and in the very advanced degrees of poisoning, where there is gangrene, that ice does harm. In many cases it diminishes the violence of the attack.

With reference to steam, it was first recommended, I think, by Mr. Prosser James, of London. Afterward it was pointed out by Oertel that steam must cure almost every case, and that it was the only remedy of any value at all, because the effect is to separate the false membrane from the mucous membrane. The fact is that when a certain point in the disease has been reached, when the false membrane is beginning to separate, steam is useful. At that time its effect is admirable. In the early stages I do not think it does any good. I think it lowers the vitality of the tissues, and that its effect is most prejudicial; but when the false membrane shows evidences of separating from the mucous membrane its effect is most beneficial. So you need have no fear of clashing heat and cold, for you use ice at first and steam afterward, when the disease has reached a certain stage. One great advantage of steam is that you can use some antiseptic with it, such as carbolic acid, salicylic acid, or any other substance you may choose. And I should advise you to use some mild antiseptic at this stage of the disease, because a certain amount of gangrene is usually present.

